# Current Controversies in Radiofrequency Ablation Therapy for Barrett’s Esophagus

**DOI:** 10.1007/s11938-016-0080-4

**Published:** 2016-02-19

**Authors:** Kamar Belghazi, Ilaria Cipollone, Jacques J. G. H. M. Bergman, Roos E. Pouw

**Affiliations:** Academic Medical Center, Department of Gastroenterology and Hepatology, Room C2-329, Meibergdreef 9, 1105 AZ Amsterdam, The Netherlands

**Keywords:** Barrett’s esophagus, Treatment, Dysplasia, Radiofrequency ablation

## Abstract

Barrett’s esophagus (BE) is the most important risk factor for esophageal adenocarcinoma. Through the sequence of no dysplasia to low-grade dysplasia (LGD) and high-grade dysplasia (HGD), eventually early cancer (EC) may develop. The risk of neoplastic progression is relatively low, 0.5–0.9 % per patient per year. However, once diagnosed, esophageal adenocarcinoma is often irresectable, and 5-year survival is only 15 %. Therefore, non-dysplastic BE patients are kept under endoscopic surveillance to detect early neoplasia in a curable stage. In case of LGD confirmed by an expert pathologist, risk of neoplastic progression is high. In these confirmed LGD patients, prophylactic ablation using radiofrequency ablation (RFA) of the Barrett’s segment has proven to significantly reduce risk of neoplastic progression. Once patients are diagnosed with HGD or EC, they have a clear indication for endoscopic treatment. The cornerstone for endoscopic management of early Barrett’s neoplasia is endoscopic resection of mucosal abnormalities. Endoscopic resection (ER) provides a large tissue specimen for accurate histological evaluation to select those patients for further endoscopic management, who have neoplasia limited to the mucosa, well to moderately differentiated and without lymph-vascular invasion. After ER, the remainder of the Barrett’s mucosa can be eradicated with RFA, to prevent occurrence of metachronous lesions.

## Introduction

Radiofrequency ablation (RFA) has proven safe and effective for eradication of Barrett’s esophagus (BE) with different grades of dysplasia. RFA is nowadays considered the treatment of choice for eradication of flat dysplastic BE or residual BE after endoscopic resection (ER) of visible lesions. This chapter will discuss the results of studies on RFA for BE, as well as current controversies in RFA treatment for BE.

## Radiofrequency Ablation Regimens

**Fig. 1:**
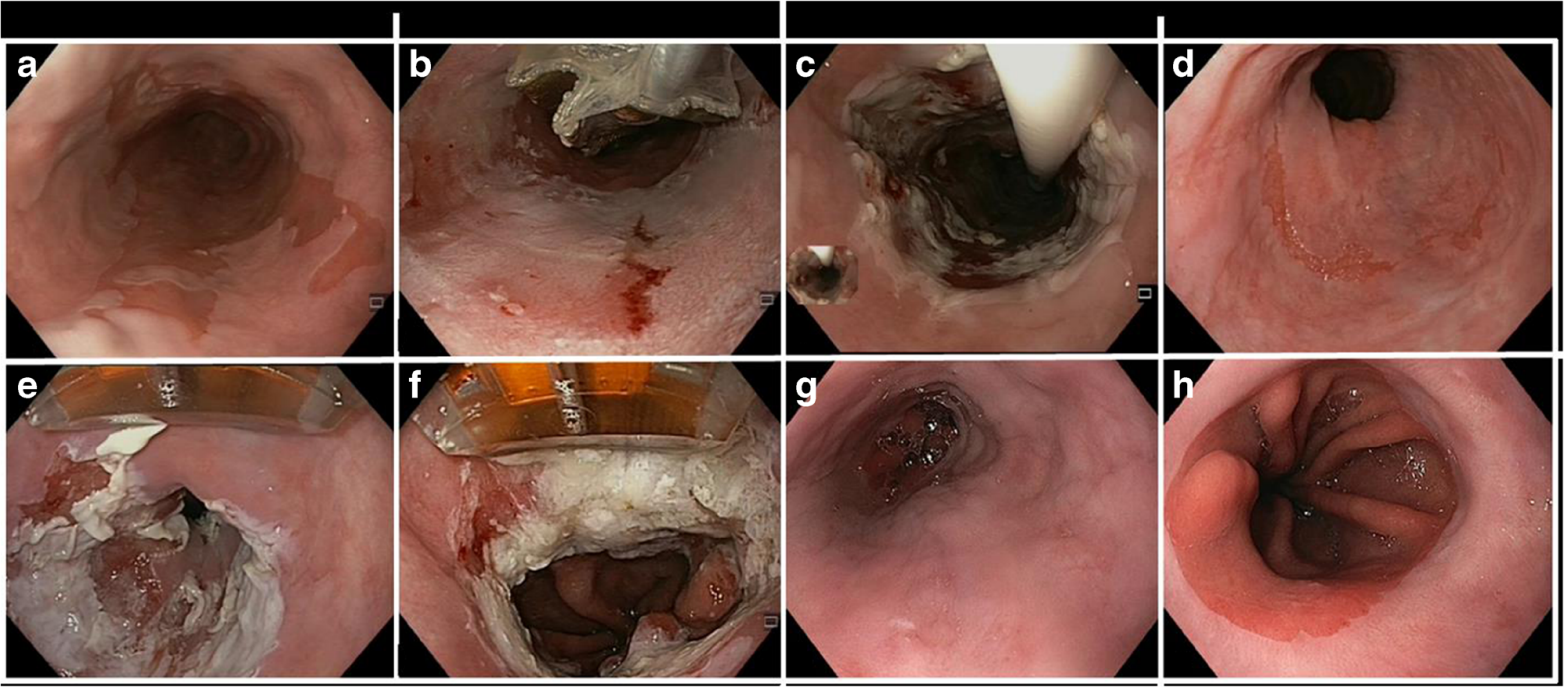
Endoscopic images of radiofrequency ablation using the Barrx^360^ system and the Barrx^90^ catheter. **a** C5M6 Barrett’s esophagus with high-grade dysplasia. **b** Circumferential ablation using the Barrx^360^ catheter. Effect immediately after the first ablation. **c** Ablation effect after the second ablation. **d** Residual Barrett’s islands 3 months after circumferential RFA. **e** Focal ablation of the residual Barrett’s epithelium using the Barrx^90^ catheter. **f** Circumferential ablation of the gastro-esophageal junction using the Barrx^90^ catheter. **g** Complete surface regression of Barrett’s epithelium. **h** Appearance of the neo gastro-esophageal junction.

RFA of BE generally starts with a stepwise circumferential ablation procedure. A standard procedure consists of sizing the esophageal inner diameter (EID) at multiple levels using a sizing catheter. Then, an ablation balloon catheter with the appropriate diameter is selected, and the entire length of the BE is ablated (Fig. 1). The Barrx^360^ ablation balloon is available in five different sizes (18–31 mm). Patients undergo a follow-up endoscopy twelve weeks after the first circumferential ablation treatment, and additional circumferential ablation is carried out if there is residual circumferential BE measuring more than 2 cm, multiple islands, or tongues of BE. Otherwise, focal ablation of residual Barrett’s mucosa and the gastro-esophageal junction (GEJ) is performed using the Barrx^90^ device (Fig. 1). The Barrx^90^ device consists of an electrode array of 20 mm mounted on an articulated platform. The Barrx^360^ device and the Barrx^90^ device are used in combination with the Barrx Flex system (GI solutions Covidien/Medtronic, Sunnyvale, CA, USA). The electrodes are designed to deliver uniform bipolar radiofrequency energy to the tissue resulting in a controlled ablation depth of 500–1000 μm.

### Circumferential Ablation

Two different ablation regimens for circumferential ablation are currently in use.

The standard regimen, consisting of two applications of 12 J/cm^2^ with a cleaning phase in between, is the most widely used and has been studied extensively [1, 2]. This regimen has the disadvantage of requiring several introductions of the scope and ablation catheter, which results in discomfort for the patient and long procedures.

A recent randomized study showed that a simplified regimen that consists of two consecutive applications of energy at 12 J/cm^2^ without a cleaning phase is easier and faster but equally effective compared to the standard regimen [3••]. The endoscopically visual regression of BE epithelium at 3 months was 88 % for the simplified regimen versus 83 % for the standard regimen. The total procedure duration was significantly shortened from 39 to 25 min. There was no difference in complete eradication of dysplasia (CE-D) [100 % for both regimens] and complete eradication of intestinal metaplasia (CE-IM) [90 % for the standard and 89 % for the simplified regimen].

Theoretically, two subsequent ablations in the same zone may result in “heat stacking” and therefore cause deeper thermal injury with subsequent stenosis. However, no RFA-related stenoses were seen in this study. Another theoretical drawback of the simplified regimen might be that it may leave small skipped BE zones un-ablated, because the same zone is ablated twice keeping the balloon in the same position. The cleaning step in the standard regimen is a good way to assess the completeness of the first ablation pass and allows ablation of skipped zones during the second pass. Furthermore, in patients with narrowing of an ER scar or relative stenosis, the RFA balloon might migrate, which could result in skipped zones or in too much overlap between ablation zones. Therefore, the standard regimen should be preferred in patients with a complex or tortuous esophagus, but we recommend using the simplified regimen in patients with an uncomplicated BE.

### Focal RFA

Focal ablation consists of two double applications of 15 J/cm^2^ with a cleaning phase in between.

A randomized trial compared the standard focal ablation with a simplified regimen consisting of three ablations of 15 J/cm^2^ without a cleaning phase in between [4]. In this study, pairs of Barrett’s areas in patients were randomized to ablation with the standard or with the simplified regimen. The results suggested that the simplified regimen is non-inferior to the standard regimen. The most important limitation of this study was that it only compared both regimens for BE islands during one procedure, whereas in clinical practice, focal ablation is also used to ablate larger areas of BE mucosa, the circumference of the GEJ, and often more than one focal procedure is required to eradicate all Barrett’s mucosa.

More recently, Künzli et al. published a retrospective analysis of a prospective cohort of 83 patients treated with the simplified protocol during all focal RFA procedures [5]. CE-D was achieved in 100 % and CE-IM in 92 % of patients after a median follow-up of 16 weeks. Although the treatment was effective, the high complication rate raised some concern about the safety of the simplified protocol. In 11 % (9/83) of patients, a stenosis requiring dilation occurred. In five out of these nine patients, the stenosis occurred at the level of the GEJ. A possible explanation could be that heat is stacked in the ablated area when three immediately subsequent ablations (15 J/cm^2^), without in-between cleaning, are performed, causing deeper injury. No long-term follow-up data of the simplified focal regimen are available yet.

Overall, the simplified regimen for focal RFA seems to be effective and practical for the endoscopist. Furthermore, it may reduce patient discomfort since less introductions of the scope and ablation catheter are necessary. However, concern remains about the stenosis rate when using the simplified regimen. A compromise might be lowering the energy settings from 15 to 12 J/cm^2^ while adhering to a simplified ablation regimen (3 × 12 J/cm^2^, no cleaning). This regimen is currently being studied in a randomized setting.

## New Ablation Tools

Three new ablation catheters for focal ablation have been added to the Barrx FLEX system. They have not been studied as extensively as the Barrx^90^ device, and only case reports or small series are published in literature [6, 7].

### Barrx^90^ Ultra Device

The electrode array is 40-mm long and 13-mm wide, resulting in a 200 % larger electrode surface compared with the regular Barrx^90^ device. Indications for its use are short segment BE or large tongues of residual BE. To prevent potential stenosis after focal ablation, the recommended treatment regimen consists of two double applications of RF energy at 12 J/cm^2^ with a cleaning phase in between. An alternative regimen consists of three applications of 12 J/cm^2^.

### Barrx^60^ Device

The electrode array is 15-mm long and 10-mm wide. As a result, the active electrode surface area is 60 % of the surface area of the Barrx^90^ device. Indications are small islands of BE in the presence of a stenosis or other challenging anatomy [6]. The recommended treatment regimen consists of two double applications of energy at 15 J/cm^2^ with a cleaning phase in between or three applications of 12 J/cm^2^.

### Channel RFA Device

The channel device is a through-the-scope device and fits through the working channel of an endoscope with a recommended diameter of 2.8 mm or larger. The design of the shaft provides catheter maneuverability, and the translucence of the device provides visibility. The electrode array has approximately the same active electrode surface area as the Barrx^60^ device. It results in very effective targeted ablation of islands in the tubular esophagus, but circumferential ablation of the GEJ is more complicated.

## Efficacy of RFA Treatment for Barrett’s Esophagus

The efficacy of RFA treatment for BE has been studied extensively. Studies to date show CE-IM rates ranging from 54 to 100 % and CE-D rates ranging from 80 to 100 % for dysplastic BE as well as for non-dysplastic BE (NDBE) patients [1, 2, 8–17, 18••, 19••]. Recently, several groups have published long-term follow-up results. A high proportion of patients achieving CE-IM and CE-D remains free of recurrence with sustained CE-IM rates reported to be between 77 and 92 % and sustained CE-D ranging from 94 to 98 % after a median follow-up duration varying from 24 to 61 months [15–17, 18••, 19••, 20–22].

It can be concluded that RFA is effective in eradicating BE with different grades of dysplasia. However, it is striking that the reported rates for CE-IM (range 54 to 100 %) show a great variation. The cause of this wide spread may be explained by differences in study design. Designs and outcome of the individual studies evaluating the efficacy of RFA for dysplastic and NDBE are summarized in Tables 1 and 2. Factors that might contribute to the variability in these studies are discussed below.

**Table 1:** Patient and study characteristics of papers on efficacy of RFA treatment for dysplastic and non-dysplastic Barrett’s esophagus

Lead. author + year	Study period	Study design	Setting	Inclusion	No. of included patients	ER (%)	Worst histological diagnosis prior to RFA (%)	PA revision
Fleisher 2008 [8] (extension of AIM II trial)	2004–2007	Prospective cohort	Multicenter, 8 academic and community hospitals, US	- Non-dysplastic BE - BE length 2–6 cm (mean 3 cm)	62	0	NDBE (100)	NS
Ganz 2008 [9]	2004–2007	Retrospective cohort	Multicenter, 16 academic and community hospitals, US	- Dysplastic BE: HGD - any BE length (median 6 cm)	92	26	HGD (100) after ER	Revision by a second expert GI pathologist
Gondrie 2008 [1] (AMC I)	2005	Prospective cohort	Single tertiary referral center, the Netherlands	- Dysplastic BE: HGD, EC - BE length 2–10 cm (median 5 cm)	11	55	LGD (18), HGD (82) after ER	Revision by a second expert GI pathologist
Gondrie 2008 [2] (AMC II)	2005–2006	Prospective cohort	Single tertiary referral center, the Netherlands	- Dysplastic BE: HGD, EC - BE length 2–10 cm (median 7 cm)	12	58	LGD (8), HGD (92) after ER	Revision by a second expert GI pathologist
Shaheen 2009 [10] (AIM dysplasia)	NS	Sham controlled randomized trial	Multicenter, 19 tertiary referral centers, US	- Non nodular dysplastic BE: LGD, HGD - BE length ≤8 cm (mean 5 cm)	84	0	LGD (50), HGD (50)	Revision by a second pathologist
Sharma 2009 [11]	2006–2007	Prospective cohort	Single tertiary referral center, US	- Dysplastic BE: LGD, HGD - Any BE length (mean 5 cm)	63	3	LGD (62), HGD (38) after ER	Revision by a second expert GI pathologist
Lyday 2010 [12]	2004–2008	Retrospective cohort	Multicenter, 4 community hospitals, US	- Non-dysplastic BE - Dysplastic BE: IND, LGD, HGD - Any BE length (median 3 cm)	137 (efficacy cohort)	NS for efficacy cohort 1.7 % in safety cohort (*n* = 429)	NDBE (80), IND (3), LGD (10), HGD (7) NS if histological grades are prior or after ER	Revision by two independent pathologists
Pouw 2010 [13] (EURO I)	NS	Prospective cohort	Multicenter, 3 tertiary referral centers, Europe	- Dysplastic BE: HGD, EC - BE length ≤12 cm (median 8 cm)	24	96	NDBE (12), LGD (46), HGD (42) after ER	Revision by a second expert GI pathologist
Van Vilsteren 2011 [14] (AMC IV)	2006–2008	Randomized clinical trial	Multicenter, 3 tertiary referral centers, Europe	- Dysplastic BE: HGD, EC - BE length ≤5 cm (median 4 cm)	22	82	NDBE, LGD, HGD After ER Numbers per histological grade NS	Revision by a second expert GI pathologist
Gupta 2013 [15]	2003–2011	Prospective cohort	Multicenter, 3 tertiary referral centers, US	- Non-dysplastic BE - Dysplastic BE: LGD, HGD, EC - BE length ≥1 cm (mean 4 cm)	592	55	NDBE (14), LGD (15), HGD (60), EC (11) NS if histological grades are prior or after ER	Revision by expert GI pathologists
Haidry 2015 [16] (UK RFA registry)	2008–2013 subgroups: 2008–2010 2011–2013	Prospective cohort	Multicenter, 25 local specialist centers, UK	- Dysplastic BE: HGD, EC - Any BE length 2008–2010: mean 6 cm 2011–2013: mean 5 cm	Total 508 2008–2010 266 2011–2013 242	Total 53 2008–2010 48 2011–2013 60	LGD (3), HGD (73), EC (24) NS if histological grades are prior or after ER	Revision by a second expert GI pathologist
Pasricha 2014 [17] (USA RFA registry)	2007–2011	Retrospective cohort	Multicenter, 148 academic and community hospitals, US	- Non-dysplastic BE - Dysplastic BE: IND, LGD, HGD, EC - Any BE length (mean 4 cm)	3169 Baseline characteristic based on durability cohort (*n* = 1634)	13	NDBE (41), IND (7), LGD (20), HGD (25), EC (7) after ER	NS
Phoa 2014 [18••]	2007–2011	RCT	Multicenter, 9 tertiary referral centers, Europe	- Dysplastic BE (expert confirmed LGD) - Any BE length (median 4 cm)	68	0	LGD (100)	Revision by an expert pathology panel
Phoa 2015 [19••]	2007–2010 (inclusion period)	Prospective cohort	Multicenter, 13 tertiary referral centers, Europe	- Dysplastic BE: HGD, EC - BE length 2–12 cm (median 6 cm)	132	90	NDBE (39), LGD (34), HGD (27) after ER	Revision by an expert pathology panel

*BE* Barrett’s esophagus, *EC* early carcinoma, *ER* endoscopic resection, *HGD* high-grade dysplasia, *IND* indefinite for dysplasia, *LGD* low-grade dysplasia, *NDBE* non-dysplastic Barrett’s esophagus, *NS* not specified, *RFA* radiofrequency ablation

**Table 2:** Treatment characteristics and outcome of papers on efficacy of RFA treatment for dysplastic and non-dysplastic Barrett’s esophagus

Lead. author + year	Treatment protocol	- Number of RFA sessions - Percentage of patients receiving escape therapy	Definition of complete eradication	CE-IM (%)	CE-D (%)	Adverse events (%)
Fleisher 2008 [8]	- Circumferential (2 × 10 J/cm^2^) max two sessions After 12 months patients enrolled in study extension: - Focal ablation (2 × 2 12 J/cm^2^) with ablation *z*-line when irregular appearance max three sessions	- RFA sessions circumferential: mean 1.5 focal: mean 1.9 - Escape therapy: NS	All biopsies negative for IM (biopsies from distal to GEJ and stomach excluded) Biopsies obtained (30 months after first ablation) from: - Neo-squamous epithelium (4Q Bx/1–2 cm)	98	–	Bleeding: 1.4 Laceration: 1.4
Ganz 2008 [9]	- Circumferential (2 × 12 J/cm^2^) max two sessions	- RFA sessions circumferential: median 1 - Escape therapy: NS	All biopsies negative for any dysplasia (CE-D) or for IM (CE-IM) Biopsies obtained (after median of 12 months (IQR 8–15) after first ablation) from: - Neo-squamous epithelium (4Q Bx/1–2 cm)	54	80	Stricture: 0.4
Gondrie 2008 [1]	- Circumferential (2 × 12 J/cm^2^) max two sessions - Focal ablation (2 × 2 12–15 J/cm^2^) with ablation *z*-line when irregular appearance max three sessions	- RFA sessions circumferential: 2 focal: median 2 - Escape therapy ER: 9 %	All biopsies negative for any dysplasia (CE-D) or for IM (CE-IM) including biopsies distal to GEJ Biopsies obtained (2 months after last treatment) from: - Neo-squamous epithelium (4Q Bx/1 cm) - Distal to GEJ	100	100	0
Gondrie 2008 [2]	- Circumferential (2 × 12 J/cm^2^) max two sessions - Focal ablation (2 × 2 12 J/cm^2^) with ablation *z*-line when irregular appearance max three sessions	- RFA sessions circumferential: median 1 focal: median 2 - Escape therapy ER: 8 %	All biopsies negative for any dysplasia (CE-D) or for IM (CE-IM) including biopsies distal to GEJ Biopsies obtained (2 months from last treatment) from: - Neo-squamous epithelium (4Q Bx/1 cm) - Distal to GEJ	100	100	Stricture: 8
Shaheen 2009 [10]	- Circumferential (2 × 12 J/cm^2^) - Focal ablation (2×, energy setting NS) ablation *z*-line not reported Total max four sessions	- RFA sessions total: mean 3.5 - Escape therapy: NS	All biopsies negative for any dysplasia (CE-D) or for IM (CE-IM) Biopsies obtained (12 months from first treatment) from: - Neo-squamous epithelium (4Q Bx/1 cm)	Total 77 HGD 74 LGD 81	Total 86 HGD 81 LGD 91	Stricture: 6 Bleeding: 1.2
Sharma 2009 [11]	- Circumferential (2 × 12 J/cm^2^) - Focal ablation (2 × 2 12 J/cm^2^) ablation *z*-line not reported max no. of sessions: NS	- RFA sessions circumferential: median 1 focal: median 1 - Escape therapy ER: 5 %	All biopsies negative for any dysplasia including biopsies distal to GEJ (CE-D). All biopsies negative for IM (CE-IM), IM distal to GEJ not considered as failure Biopsies obtained (median 21 months from first treatment) from: - Neo-squamous epithelium (4Q Bx/1 cm) - Distal to GEJ	Total 79 HGD 67 LGD 87	Total 89 HGD 79 LGD 95	Stricture: 1.6 Bleeding: 1.6
Lyday 2010 [12]	- Circumferential (2 × 10–12 J/cm^2^) - Focal ablation (2 × 2 12 J/cm^2^) ablation *z*-line not reported max no. of sessions: NS	- RFA sessions total: mean 2.1 - Escape therapy: NS	All biopsies negative for any dysplasia or IND (CE-D) or for IM (CE-IM) Biopsies obtained (median 20 months (IQR: 17–26) from first treatment) from: - Neo-squamous epithelium (4Q Bx/1–2 cm)	Total 77 dysplastic 78 NDBE 76	Total 100 dysplastic 100 NDBE –	NS for efficacy cohort Based on safety cohort (*n* = 429): Stricture: 2 Bleeding: 0.9 Laceration: 0.2
Pouw 2010 [13]	- Circumferential (2 × 12 J/cm^2^) max two sessions - Focal ablation (2 × 2 12 J/cm^2^) with standard circumferential ablation of *z*-line max three sessions	- RFA sessions circumferential: median 1 focal: median 1 - Escape therapy ER: 8 %	All biopsies negative for any dysplasia (CE-D) or for IM (CE-IM) including biopsies distal to GEJ Biopsies obtained (2 months after last treatment) from: - Neo-squamous epithelium (4Q Bx/1 cm) - Distal to GEJ	96	100	Stricture: 4 Laceration: 21
v. Vilsteren 2011 [14]	- Circumferential (2 × 12 J/cm^2^) - Focal ablation (2 × 2 15 J/cm^2^) with standard circumferential ablation of *z*-line max two sessions (≤2 circumferential)	- RFA sessions total: median 3 - Escape therapy ER: 5 % APC: 5 % hot biopsy forceps: 9 %	All biopsies negative for any dysplasia or IND (CE-D) or for IM (CE-IM) including biopsies distal to GEJ Biopsies obtained (median 8 months (IQR 5–10) from first treatment) from: - Neo-squamous epithelium (4Q Bx/1 cm) - Distal to GEJ	96	96	Stricture: 14 Bleeding: 14 Laceration: 5
Gupta 2013 [15]	- Circumferential (2 × 10–12 J/cm^2^) - Focal ablation (2 × 2 10–12 J/cm^2^) with standard circumferential ablation of *z*-line Ablation 2–3-month intervals until histologic and endoscopic remission	- RFA sessions 1 session: 29 % of patients 2 sessions: 35 % of patients 3–10 sessions: 36 % of patients - Escape therapy: NS	All biopsies negative for any dysplasia or IND (CE-D) or for IM (CE-IM) including biopsies distal to GEJ Biopsies obtained (24 months after first treatment) from: - Neo-squamous epithelium (4Q Bx/1–2 cm) - Distal to GEJ Not all sites reported histology of neo-squamous and GEJ separately	56	NS	Stricture: 5 Bleeding: 1 Laceration:0.4
Haidry 2014 [16]	- Circumferential (2 × 12 J/cm^2^) - Focal ablation (2 × 2 15 J/cm^2^) ablation *z*-line not reported Treatment period 12 months: max four sessions In case of residual BE hereafter additional RFA or ER	2008–2010 - RFA sessions total: mean 2.6 - Escape therapy ER: 13 % 2011–2013 - RFA sessions total: mean 2.5 - Escape therapy ER: 2 %	All biopsies negative for any dysplasia or IND (CE-D) or for IM (CE-IM). IM in biopsies distal to GEJ not considered as failure Biopsies obtained at end of treatment (at 12 months) from: - Neo-squamous epithelium (4Q Bx/1 cm) - Distal to GEJ	Total 70 2008–2010 57 2011–2013 83	Total 84 2008–2010 77 2011–2013 92	Total 7.8 2008–2010 stricture: 9.4 2011–2013 stricture: 6.2
Pasricha 2014 [17]	- Circumferential (2 × 12 J/cm^2^) - Focal ablation (2 × 2) ablation *z*-line not reported max no. of sessions: NS	- RFA sessions circumferential: mean 0.7 focal: 2.2 - Escape therapy RFA: 2 %	All biopsies negative for IM (CE-IM) Biopsies obtained (>12 months after treatment) from: - Neo-squamous epithelium (4Q Bx/1 cm)	85	–	NS
Phoa 2014 [18••]	- Circumferential (2 × 12 J/cm^2^) max two session - Focal ablation (2 × 2 12 J/cm^2^) with standard circumferential ablation *z*-line max three sessions	- RFA sessions total: median 3 - Escape therapy ER: 7 % APC: 18 %	All biopsies negative for any dysplasia (CE-D) or for IM (CE-IM) including biopsies distal to GEJ Biopsies obtained at end of treatment from: - Neo-squamous epithelium (4Q Bx/2 cm) - Distal to GEJ	88	93	Stricture: 11.8 Bleeding: 1.5 Laceration: 4.4
Phoa 2015 [19••]	- Circumferential (2 × 12 J/cm^2^) max two sessions - Focal ablation (2 × 2 12 J/cm^2^) with standard circumferential ablation of *z*-line max three sessions	- RFA sessions Circumferential: median 1 Focal: median 2 - Escape therapy ER (for lesions >5 mm): 7 % APC (for lesions <5 mm): 11 %	All biopsies negative for any HGD or EC (CE-N) or for IM (CE-IM) including biopsies distal to GEJ Biopsies obtained (at median 12 months (IQR 9–19) from first treatment) from: - Neo-squamous epithelium (4Q Bx/2 cm) - Distal to GEJ	93	98	Stricture: 6 Laceration: 8 Bleeding: 1

*APC* argon plasma coagulation, *BE* Barrett’s esophagus, *Bx* biopsies, *CE*-*D* complete eradication of dysplasia, *CE*-*IM* complete eradication of intestinal metaplasia, *EC* early carcinoma, *ER* endoscopic resection, *FU* follow-up, *GEJ* gastro-esophageal junction, *HGD* high-grade dysplasia, *IND* indefinite for dysplasia, *LGD* low-grade dysplasia, *NDBE* non-dysplastic Barrett’s esophagus, *NS* not specified, *RFA* radiofrequency ablation

### Study Design

Retrospective studies report CE-IM rates ranging from 54 to 85 % [9, 12, 17]. The strict treatment and follow-up protocols often used in studies with a prospective design might result in higher eradication rates compared with results from retrospective studies. However, by excluding studies with a retrospective design, a great variation in CE-IM rates is still observed (56 to 100 %).

Furthermore, the research setting in which the study is conducted might influence the outcome of the study. The majority of the studies were conducted in tertiary referral centers [1, 2, 10, 11, 13–15, 18••, 19••]. The higher exposure to BE patients in these tertiary referral centers could lead to higher eradication rates at these centers. One should therefore be careful with extrapolating these results to common practice. However, no obvious difference was seen when the results of studies conducted in tertiary referral centers were compared to studies from community and academic hospitals (range 56 to 100 % vs 54 to 98 %).

### Patient Selection

Efficacy of RFA has been studied in dysplastic BE patients as well as in NDBE patients. The AIM trial conducted by Fleisher et al. included exclusively NDBE patients. At 30-month follow-up, CE-IM was found in 98 % of patients. Of these patients, 92 % remained free of IM during the 60-month follow-up [8, 20]. Most studies included patients with dysplastic BE at baseline. Three studies included both dysplastic and NDBE patients, and two of these studies reported separate results for the subgroups based on baseline histology [12, 15, 17]. Gupta et al. found no association between entry histology and achieving CE-IM in their large prospective cohort study. However, Pasricha et al. found in their prospective cohort that more advanced pre-treatment histology was associated with an increased yearly recurrence rate when using Kaplan-Meier analysis [17]. This was confirmed in a recent meta-analysis by Orman et al. evaluating the efficacy and durability of RFA treatment. They included 18 studies reporting on efficacy (*n* = 3801) and pooling of the data resulted in a CE-IM rate of 78 % and a CE-D rate of 91 % [23]. The authors found that efficacy differed based on entry histology, with a trend toward reduced efficacy for patients with a more advanced degree of dysplasia. The reason for this relationship is not entirely clear since RFA results in a uniform depth of ablation. The authors proposed a number of possible explanations for this finding. Because longer BE lengths are associated with progression to advanced histology [24], histology might be a surrogate marker of BE length. Another explanation could be that high-grade dysplasia (HGD) is possibly more likely to harbor malignancy compared with low-grade dysplasia (LGD) or NDBE and that HGD might therefore also be more likely to penetrate beyond the depth of ablation. Finally, the authors mention that more advanced histology might be less susceptible for thermal ablation techniques compared with non-dysplastic Barrett’s epithelium [23].

Several included studies reported an association between BE length and outcome [12, 15, 17]. Orman et al. hypothesized that CE-IM rates are lower in patients with longer BE segments because the risk of missing a portion of Barrett’s epithelium during treatment is increased in these patients because of the larger surface that has to be ablated [23].

Studies with a BE length restriction showed CE-IM ranging from 77 to 100 % [1, 2, 8, 10, 13, 14, 19••]. Whereas studies that included all BE lengths reported CE-IM rates between 54 and 88 %. However, the median reported BE length did not differ a lot between these two groups. The studies with a BE length restriction had a median BE length between 3 and 8 cm, and the studies analyzing any BE length reported a median BE length ranging from 3 to 6 cm [9, 11, 12, 15–17, 18••].

In a prospective cohort study by Alvarez Herrero et al., 26 dysplastic BE patients with a BE length of ≥10 cm were analyzed [25]. The data showed that RFA for longer BE segments resulted in CE-IM and CE-D of 79 and 83 %, respectively. In 15 % of the cases, RFA treatment was discontinued because of poor healing and no regression. Since these patients were considered as failure for CE-IM and CE-D, this resulted in slightly lower total efficacy rates. The authors concluded that RFA for BE segment ≥10 cm is effective but more challenging.

### Treatment Protocol

Because the focal ablation device became available after the circumferential device, earlier studies focused on outcomes of circumferential RFA alone. In the retrospective cohort study by Ganz et al., 92 patients were treated with a maximum of two circumferential ablation sessions (median 1). Respectively, 52 and 80 % of patients achieved CE-IM and CE-D after a median of 12-month follow-up after the first treatment session [9]. The remaining studies used a stepwise approach of circumferential and focal RFA, as well as combining endoscopic mucosal resection with RFA.

Several studies have demonstrated that the GEJ is the area most at risk for recurrence of neoplasia [14, 26–28]. Since endoscopic differentiation between gastric mucosa and IM is very difficult, we believe that the GEJ should always be circumferentially treated. Because the balloon catheter does not make optimal contact with the mucosa at the level of the GEJ, we advise performing circumferential ablation with the focal device (Fig. 1). Standard circumferential ablation of the GEJ during focal ablation was performed in five studies [13–15, 18••, 19••]. The majority of studies did not perform standard ablation of the GEJ. Of these studies, three ablated the GEJ when it had an irregular appearance [1, 2, 8], and five did not perform ablation of the GEJ at all [9–12, 15, 20]. The latter group of studies showed CE-IM rates between 54 and 85 %.

In most studies, ER was performed prior to RFA treatment [1, 2, 9, 11–17, 20]. Haidry et al. examined prospective data from the UK RFA registry [16]. Five hundred and eight dysplastic Barrett patients were treated between 2008 and 2013, 70 % achieved CE-IM and 84 % achieved CE-D at the end of the treatment period. Based on the time period patients were treated in, two subgroups were created (2008–2010 and 2011–2013). Subgroup analysis showed that outcome for patients treated in the later time period improved significantly compared with that for patients treated in the former period (CE-D 92 vs 77 % and CE-IM 83 vs 57 %). The percentage of ER before RFA increased from 48 % in the former time period to 60 % in the later time period. The increased use of ER prior to RFA resulted in a decrease of escape ER during RFA treatment (13 vs 2 %). The more widely used ER prior to RFA treatment could be an explanation for the improved clinical outcome over time according to the authors. By eradicating all visible and nodular neoplasia, a flat BE segment is created resulting in better contact with the RFA balloon catheter. Therefore, treatment might be more successful after widespread ER. Furthermore, a subgroup of patients that have more advanced disease (submucosal invasion) can be filtered out after ER. These patients have less favorable outcome after endoscopic therapy resulting in overall lower outcome rates. Other factors that could have contributed to this improvement are increased physician awareness, improved disease staging, better patient selection, and improved endoscopic skills. Several other authors have evaluated the relation between ER prior to RFA and treatment outcome as well, but did not find an association [17, 21, 29].

### Biopsy Protocol

As already mentioned above, the GEJ is most at risk for recurrence of neoplasia. We advise to obtain biopsies immediately distal to the neo-GEJ as an objective endpoint of eradication of IM.

All studies included 4-quadrant biopsies from every 1 or 2 cm from the neo-squamous epithelium in their biopsy protocol. In the majority of studies, biopsies were also taken just below the neo-GEJ [1, 2, 11, 13–16, 18••, 19••]. Two of these studies did not include IM from distal to the neo-GEJ as failure of CE-IM [11, 16]. It is plausible that studies defining IM from biopsies taken distal to the neo-GEJ as failure CE-IM show lower eradication rates. However, in the reviewed studies, this tendency was not seen. Studies considering IM in all biopsies (also distal to the neo-GEJ) as failure reported CE-IM rates ranging from 56 to 100 %. Studies that only considered IM found in the neo-squamous epithelium as failure showed CE-IM rates between 54 and 98 %.

## RFA for Barrett’s Esophagus Containing Low-Grade Dysplasia

The natural history of LGD in BE remains controversial. Some studies have reported neoplastic progression rates for LGD comparable to those reported for NDBE [30–32]. However, other studies have shown that in patients with a confirmed LGD diagnosis after expert pathology review, the risk of progression to HGD or esophageal adenocarcinoma (EAC) may be as high as 27 % within 2 years and 8 months of follow-up [33, 34].

Recently, a meta-analysis found that BE surveillance cohorts with a high rate of LGD diagnosis report low rates of progression to HGD or EAC, whereas a much higher rate of neoplastic progression is reported in cohorts where LGD is diagnosed less frequently [35].

The most important issue seems to be the reliability of the baseline LGD diagnosis.

Especially in community settings, overdiagnosis of LGD results in underestimation of the risk of neoplastic progression.

Curvers et al. investigated the natural history of LGD in a large community-based cohort of BE patients. After the original histological diagnosis was reviewed by two expert gastrointestinal pathologists, the LGD diagnosis was confirmed in only 15 % of patients. The LGD diagnosis was downstaged in the remaining 85 %. The rate of neoplastic progression was 13.4 % per patient-year for patients with confirmed LGD, compared with 0.49 % per patient-year for patients who were downstaged to NDBE [36].

Furthermore, Duits et al. retrospectively examined the prognostic value of an expert pathology panel for reviewing the LGD diagnosis of community hospital pathologists [37••]. Similarly to the aforementioned study, Duits et al. demonstrated that only 27 % of the original LGD diagnoses were confirmed, while the remaining 73 % were downstaged. After a median follow-up of 39 months, the risk of progression to HGD or EAC was 9.1 % per patient-year in the confirmed LGD group. In contrast, patients with a downstaged diagnosis (to NDBE or indefinite for dysplasia) had a neoplastic progression rate of 0.6 and 0.9 %, respectively.

Endoscopic surveillance has been recommended by international guidelines for BE containing LGD. However, taking the high risk of neoplastic progression in patients with confirmed LGD in consideration, this might no longer be the adequate strategy for this group of patients.

A randomized trial by Phoa et al. compared endoscopic surveillance with prophylactic RFA in BE patients with confirmed LGD [18••]. In the control group, the rate of progression was 11.8 % per patient per year, similar to the studies mentioned above. Ablation reduced the risk of progression to HGD or EAC by 25.0 % (1.5 % for ablation vs 26.5 % for surveillance). In the ablation group, CE-D was achieved in 92.6 % of patients and CE-IM in 88.2 %. During follow-up, complete eradication was maintained in 98.4 % of cases. Adverse events occurred in 13 patients (19 %), with esophageal stricture in 11.8 %. The multivariable analysis demonstrated the following three independent predictors of progression in the control group: the number of years since the diagnosis of BE, the number of endoscopies with dysplasia prior to inclusion, and circumferential Barrett length in centimeters.

In a multicenter study based in the US, Small et al. compared the rate of progression of LGD following RFA with the rate of progression of endoscopic surveillance alone in routine clinical practice [38]. Similar results were found as in the abovementioned study. CE-D was reached in 95.6 % in the ablation group and CE-IM in 77.8 %. The annual rates of progression to HGD or EAC were 6.6 % in the surveillance group and 0.77 % in the RFA group.

These recently published papers [18••, 35, 36, 37••] resulted in revision of the NICE guideline on ablative therapy for the treatment of BE in 2014 [39]. The guideline now includes LGD as a valid indication for RFA. Furthermore, the British Society of Gastroenterology (BSG) guideline for the management of Barrett’s esophagus was recently updated with regard to the diagnosis and treatment of LGD [40]. The BSG recommends that patients with LGD should have a repeat endoscopy within 6 months. If LGD is found in any of the follow-up endoscopies and is confirmed by an expert GI pathologist, the patient should be offered endoscopic ablation therapy after review by the specialist multidisciplinary team. If ablation is not performed, 6-month surveillance is recommended.

## Post Ablation Follow-Up

Meticulous endoscopic inspection of the neo-squamous epithelium and the neo-GEJ, in order to rule out the presence of residual columnar mucosa, forms the basis for endoscopic follow-up.

This can be achieved by using high-resolution endoscopy with narrow-band imaging (NBI) performed by an endoscopist with a trained eye.

In our opinion, extensive biopsies from the neo-squamous epithelium have become obsolete given the low rate of buried glands [41]. When the esophagus is inspected in detail, it is sufficient to obtain targeted biopsies from residual columnar mucosa or visual abnormalities. If small islands (<5 mm) with columnar epithelium are detected, these can best be treated right away with argon plasma coagulation (APC) instead of obtaining biopsies since this can lead to a false-positive diagnosis of buried glands [42]. Furthermore, if the biopsies prove to be positive for dysplasia, it is often impossible to re-detect such small areas on endoscopy; therefore, immediate APC is advisable.

Obtaining biopsies immediately below the GEJ remains important, given the high risk for recurrence in this area [26–28]. A reliable endoscopic tool to predict if all Barrett’s mucosa has been eradicated at this level is not available [43]. Even endoscopic detection techniques such as NBI have not been able to aid the endoscopist in the differentiation between gastric mucosa and IM [44]. Therefore, we always obtain biopsies immediately distal (<5 mm) to the neo-GEJ as an objective endpoint for eradication of IM. The downside of this biopsy protocol is that it can lead to detection and overestimation of non-dysplastic IM in the presence of a normal appearing neo-GEJ on endoscopy.

The clinical relevance of IM in the cardia is uncertain because focal IM in this area may reflect insufficient treatment, recurrence of disease, or an irrelevant normal finding [22, 45]. If IM is found in this region, we advocate repeating focal ablation only when IM is detected at the first follow-up endoscopy; subsequent touch-up ablation during follow-up is unnecessary when dysplasia is absent.

The recommended follow-up interval depends on the initial grade of dysplasia:

### Patients with IMC/HGD

We recommend performing follow-up endoscopies at 3, 9, and 21 months after the last treatment session, then annually hereafter. If there is sustained CE-IM five years after treatment, surveillance intervals can be extended or surveillance can be stopped also taking into consideration the patients age and general condition. Others perform surveillance endoscopies every three months for the first year, every six months for the second year, and annually thereafter [46].

### Patients with LGD/NDBE

We recommend performing follow-up endoscopies at 3, 9, and 21 months after the last treatment session, then annually hereafter. If there is sustained CE-IM three years after treatment, surveillance can be stopped or intervals prolonged. The published literature on the durability of RFA shows that the risk of progression in these patients is small [23].

## Future Perspectives in RFA Treatment

Recent developments in endoscopic techniques have significantly improved patient care over the past years. Different ablation regimens have been studied leading to simplified ablation regimens. Circumferential ablation is cheaper and faster, but equally safe and effective, when the cleaning phase between ablations is omitted.

Recently the Barrx^360^ Express RFA balloon catheter (Express 360) has been developed. The Express 360 is a circumferential balloon catheter, which contains a 4-cm long bipolar electrode that is wrapped around a balloon and that features the ability to self-adjust to the inner esophageal lumen. Therefore, the Express 360 may adjust for differences in EID over the length of the ablation zone. Furthermore, circumferential ablation using the Express 360 may result in shorter procedure duration and decreased patient discomfort since fewer introductions of the endoscope and catheters are necessary. The Express 360 is currently being studied in a randomized trial.

Different ablation regimens for focal ablation have been studied as well. However, there is still controversy about the ideal energy settings for focal ablation. In Europe, the focal device has been mainly used at 15 J/cm^2^, both for the standard and the simplified regimen. Lowering the energy density to 12 J/cm^2^ (in accordance to the US standard protocol) when using the simplified triple application may reduce the risk of fibrosis and stenosis. A randomized clinical trial comparing the standard regimen (2 × 15 J/cm^2^—clean—15 J/cm^2^) to the simple triple regimen (3 × 12 J/cm^2^—no clean) is currently being conducted.
